# Supplementary material for: Comparison of the most likely low-emission electricity production systems in Estonia
Source: PLoS One. 2021 Dec 30;16(12):e0261780. doi: 10.1371/journal.pone.0261780 (PMC8717974; doi:10.1371/journal.pone.0261780)
Supplement: Attachment — Submitted filename: response to reviewers.docx [file pone.0261780.s001.docx]

**Response to reviewers**

**Reviewer 1**

*Please include some discussion on the use of the DTR system for improving the transmission capacity of the power systems and provide a descriptive comparison on how this DTR system is beneficial?*

Although dynamic thermal ratings is an interesting and important topic, it is only one of the many potential technologies and techniques that will be needed in a grid with large amounts of wind and solar. The grid improvement costs, which are the cost component that could be reduced with DTR, are only a minor factor in the overall system cost that was the focus of this article, accounting for only about 1-2 EUR/MWh. The impact of DTR on the overall system cost would be even smaller because some grid improvements cannot be avoided using DTR. The current article takes a higher-level approach and focuses on the Estonian electricity system as a whole, and therefore, including a discussion of all potential technologies and implementation details is beyond the scope of our current article. We do, however, thank the reviewer for the informative references and may find them useful in our future work.

**Reviewer 2**

*All factors and parameters with the respective impact should be highlighted again after all analysis have been done. A table of graph can be included to strengthen your findings.*

We have now included a sensitivity analysis that investigates the impact of all the variable parameters used in the model. Figure 8 gives an overview of this analysis and a new subsection was added to discuss the results.

*I am not satisfy with too many references. The authors should be focused on recent journals (5 years back).*

It is true that the article has many references, but each of these references are important to the article and no unnecessary references have been included. One reason there are so many references is because this study required estimates for many different parameters used in modeling the electricity system in Estonia.

We have also used the most recent studies we could find that give the information we needed. A large portion of the references are actually to literature published within the past 5 years. Of the remaining references, the majority have at least been published within the last 10 years. For some data and information, it was not possible to find more recent sources. Additionally, the underlying goal when selecting literature is to find the most reliable information. Although more recent literature may be, on average, more likely to be reliable, this is not always the case and sometimes citing an older, but more reliable, study is best. This is one reason we have cited some sources that are older than 10 years: they are key studies in the field and provide reliable data. One such example is the IPCC report on carbon capture published in 2005.

For these reasons, we have kept the list of cited works the same, but if the reviewer sees specific references that could be removed or replaced with more recent studies, we would be willing to take those suggestions.

**Editor**

*Please ensure that your manuscript meets PLOS ONE's style requirements, including those for file naming.*

We have renamed the figure files and reformatted the article to meet PLOS ONE's style guidelines to the best of our knowledge.

*We note that the grant information you provided in the ‘Funding Information’ and ‘Financial Disclosure’ sections do not match.*

We have updated this information so it matches.

*Funding information should not appear in the Acknowledgments section or other areas of your manuscript.*

This was removed.

*Please ensure that you refer to Figure 2 in your text as, if accepted, production will need this reference to link the reader to the figure.*

Thank you for noticing this error. We had simply made a mistake and referenced Figure 1 in the spot where Figure 2 should have been referenced. We have corrected this.

*Please review your reference list to ensure that it is complete and correct.*

We have reviewed our list of references and to the best of our knowledge all are correct.
