# Supplementary material for: Comparison of the most likely low-emission electricity production systems in Estonia
Source: PLoS One. 2021 Dec 30;16(12):e0261780. doi: 10.1371/journal.pone.0261780 (PMC8717974; doi:10.1371/journal.pone.0261780)
Supplement: Attachment — Submitted filename: response to reviewers.docx [file pone.0261780.s002.docx]

**Response to reviewers**

**Reviewer 1**

*The authors presented a study on comparing the electrical systems of Estonia. However, I think the following can help to improve the quality of the paper further 1) Please include some discussion on the use of the DTR system for improving the transmission capacity of the power systems and provide a descriptive comparison on how this DTR system is beneficial?*

We have included the following discussion of DTR systems in the revised version of the article:

"One such example for increasing the performance of existing lines is dynamic thermal rating. The capacity of transmission lines is limited to ensure that the temperature of the lines does not exceed their rated value. Currently, a fixed, static thermal rating is generally used, but by dynamically adjusting the thermal rating based on actual environmental conditions, the capacity of the lines could be increased during some periods."

To round out the discussion and improve the flow of the text, we included these sentences in a larger paragraph that discusses this and a few other proposed strategies that could be used to improve the flexibility and reduce the cost of grids with a high penetration of renewables.

Earlier Reviewer 2 expressed concern about the number of references in the article. Obviously, introducing this new section increased the number of references. To also accommodate the earlier comments of Reviewer 2, we have limited the number of new references we included and gave preference to sources published within the last 5 years. And as requested in the requirements of the journal, here is a list of the new references we have added (references 168-172 in the references list):

168.	Cochran J, Miller M, Zinaman O, Milligan M, Arent D, Palmintier B, O’Malley M, Mueller S, Lannoye E, Tuohy A, Kujala B, Sommer M, Holttinen H, Kiviluoma J, Soonee SK. Flexibility in 21st Century Power Systems [Internet]. National Renewable Energy Lab. (NREL), Golden, CO (United States); 2014 May [cited 2021 Dec 8]. Report No.: NREL/TP-6A20-61721. Available from: https://www.osti.gov/biblio/1130630

169.	Jian L, Zechun H, Banister D, Yongqiang Z, Zhongying W. The future of energy storage shaped by electric vehicles: A perspective from China. Energy. 2018;154:249–57.

170.	Martinot E. Grid Integration of Renewable Energy: Flexibility, Innovation, and Experience. Annu Rev Environ Resour. 2016;41(1):223–51.

171.	Karimi S, Musilek P, Knight AM. Dynamic thermal rating of transmission lines: A review. Renew Sustain Energy Rev. 2018 Aug 1;91:600–12.

172.	Verma R. Application of Computational Techniques in Demand Response: A Review. In: 2021 7th International Conference on Advanced Computing and Communication Systems (ICACCS). 2021. p. 1242–6.
